# Elevation in Serum Concentration of Bone-Specific Alkaline Phosphatase without Elevation in Serum Creatinine Concentration Secondary to Adefovir Dipivoxil Therapy in Chronic Hepatitis B Virus Infection

**DOI:** 10.1155/2013/739247

**Published:** 2013-09-09

**Authors:** Hiroshi Abe, Nobuyoshi Seki, Tomonori Sugita, Yuta Aida, Haruya Ishiguro, Tamihiro Miyazaki, Munenori Itagaki, Satoshi Sutoh, Yoshio Aizawa

**Affiliations:** Division of Gastroenterology and Hepatology, Department of Internal Medicine, Jikei University School of Medicine Katsushika Medical Center, Katsushika-ku, Tokyo 125-8506, Japan

## Abstract

Of 168 patients with chronic hepatitis B virus (HBV) infection-related liver disease, 20 patients who had received 100 mg of lamivudine plus 10 mg/day of adefovir dipivoxil (ADV) (ADV group) and 124 patients who had received 0.5 mg/day of entecavir or 100 mg/day of lamivudine (non-ADV group) for >1 year were enrolled. For comparative analyses, 19 well-matched pairs were obtained from the groups by propensity scores. At the time of enrollment, serum creatinine and phosphate concentrations were similar between the ADV and non-ADV groups; however, urinary phosphate (*P* = 0.0424) and serum bone-specific alkaline phosphatase (BAP) (*P* = 0.0228) concentrations were significantly higher in the ADV group than in the non-ADV group. Serum BAP was significantly higher at the time of enrollment than before ADV administration in the ADV group (*P* = 0.0001), although there was no significant change in serum BAP concentration in the non-ADV group. There was a significant positive correlation between the period of ADV therapy and ΔBAP (*R* ^2^ = 0.2959, *P* = 0.0160). Serum BAP concentration increased before increase in serum creatinine concentration and was useful for early detection of adverse events and for developing adequate measures for continuing ADV for chronic HBV infection-related liver disease.

## 1. Introduction

Chronic hepatitis B virus (HBV) infection causing chronic hepatitis, cirrhosis, and hepatocellular carcinoma affects more than 350 million people worldwide [1, 2]. Antiviral therapies that contain interferon and nucleos(t)ide analogues are used in patients who are infected with HBV and are at a higher risk of developing cirrhosis and hepatocellular carcinoma [1, 3, 4]. The purpose of the treatment is to suppress HBV replication and to prevent mortality associated with disease progression to end-stage liver disease and major complications [4, 5]. Currently, 5 oral nucleos(t)ide analogues are approved for the treatment of liver disease related to chronic HBV infection in Europe and USA, although only 3 nucleos(t)ide analogues, namely, lamivudine (LAM), entecavir (ETV), and adefovir dipivoxil (ADV), are approved for use in Japan. LAM, the first approved nucleoside analogue for chronic HBV infection-related liver disease, suppresses HBV replication and improves hepatic inflammation in most patients [6]. However, more than 60% of patients with chronic HBV infection who receive long-term LAM therapy become resistant to the agent within 4 years of starting the therapy [7]. For patients with LAM resistance, virological breakthrough due to development of ADV-resistant mutations occurred in 21% of 14 patients within 18 months after changing to ADV monotherapy [8]; ETV-resistant mutations were found in 8% of 151 patients 2 years after changing to ETV monotherapy (1 mg once per day) [9]. Therefore, LAM monotherapy has not been used for patients with chronic HBV infection since ETV was approved. In patients with LAM resistance, tenofovir (TDF, not yet approved for use in Japan) or ADV therapy is advised [10].

The appearance of virological and biochemical breakthroughs during combination therapy with ADV and LAM is very rare in patients with LAM resistance; therefore, combination therapy is recommended in such cases [11, 12]. At present, combination therapy with ADV and LAM must be continued for a long period of time, even though long-term use of ADV is associated with a slight risk of renal toxicity [13, 14]. Although ADV has a favorable risk/benefit profile with little or no evidence of renal toxicity after 48 weeks of low-dose treatment (10 mg per day) [15, 16], some studies have reported development of severe osteomalacia caused by renal tubular dysfunction (Fanconi syndrome) after long-term use of ADV [17, 18]. Therefore, establishment of a protocol is required for early identification of osteomalacia caused by renal tubular dysfunction during the administration of ADV. Serum bone-specific alkaline phosphatase (BAP) is the most commonly used bone disease marker in hemodialyzed patients [19]. Serum BAP levels increase gradually in osteomalacia secondary to the administration of ADV, without increase in serum creatinine concentration [17, 18]. Therefore, serum BAP concentration may be a potentially useful marker for early detection of osteomalacia caused by renal impairment secondary to the administration of ADV.

To determine the significance of these indicators for the management of patients receiving long-term ADV plus LAM therapy, we evaluated bone metabolism markers, including serum and urinary phosphate concentrations and serum BAP, in chronic hepatitis B (CHB) patients receiving nucleos(t)ide analogues.

## 2. Materials and Methods

### 2.1. Patients

This study complies with the standards of the 1975 Declaration of Helsinki and current ethical guidelines; written informed consent was obtained from each patient.

Between August 2003 and January 2012, 168 consecutive patients positive for hepatitis B surface antigen (HBsAg), who presented to the Division of Gastroenterology and Hepatology of our institution, received nucleos(t)ide analogue therapy. Among them, 144 consecutive patients who received ADV or the other nucleoside analogues (LAM or ETV) for more than 1 year were enrolled. Most patients had HBV genotype B or C, as seen in a previous Japanese study [12]. Of these, 20 patients had received a combination of 100 mg of LAM plus 10 mg of ADV per day (ADV group), and 124 patients had received either 100 mg of LAM per day or 0.5 mg of ETV (non-ADV group). Patients in the ADV group received additional ADV because of increase in serum HBV DNA levels (≥1 log_10_ copies/mL) during LAM monotherapy. The non-ADV group included 34 patients who started LAM therapy (100 mg per day) but had been subsequently switched to ETV therapy (0.5 mg per day) to avoid the appearance of LAM-refractory HBV. This group also included 90 patients who had received 0.5 mg of ETV per day as a first-line therapy. Exclusion criteria were as follows: presence of antibodies to hepatitis C virus or HIV; a current alcohol consumption of >20 g/day; and presence of hepatocellular carcinoma, other liver diseases, progressive decompensated liver cirrhosis, or renal dysfunction at the time of starting nucleos(t)ide analogue therapy (serum creatinine: male, >1.3 mg/dL; female, >1.1 mg/dL). Patients who had hypertension [20] and/or diabetes mellitus [21] were also excluded from this study owing to the risk of renal impairment. In addition, patients who were receiving vitamin D were excluded from this study because this therapy may affect bone turnover. All blood samples were obtained from patients after fasting. 

To reduce the confounding effects of covariates, we used propensity scores [22, 23] to match the ADV group to the non-ADV group during nucleos(t)ide analogue therapy based on the stage of liver disease at the time of enrollment and renal function at the time of receiving the nucleos(t)ide analogue. Variables that may have influenced the treatment outcomes, including age, sex, the duration of nucleos(t)ide analogue therapy, platelet count, serum alanine aminotransferase (ALT), serum albumin at the time of enrollment (nucleos(t)ide analogue therapy for >1 year), and serum creatinine level at the time of receiving nucleos(t)ide analogues, were used to generate a propensity score ranging from 0 to 1 by logistic regression. The nearest available match on the estimated propensity score was used to select participants in the ADV group and find participants in the non-ADV group with the closest propensity scores. Nineteen well-matched pairs of patients in the ADV and non-ADV groups were obtained.

### 2.2. Analysis of Serological and DNA Markers for HBV

HBsAg in patients' sera was tested by enzyme immunoassay using commercially available kits (Dainabott, Tokyo, Japan). The serum HBV DNA concentration was monitored using a polymerase chain reaction assay (COBAS Amplicor HBV monitor test, Roche Diagnostics K. K., Tokyo, Japan; lower limit of detection, 2.6 log copies/mL) before November 2007 and by another polymerase chain reaction assay (COBAS AmpliPrep-COBAS Taqman HBV Test, Roche Diagnostics K. K.; lower limit of detection, 2.1 log copies/mL) after December 2007. YMDD mutations were detected using a line probe assay (INNO-LiPA HBV DR assay, Innogenetics NV).

### 2.3. Examination of Bone Turnover Markers

Serum BAP was measured at the time of enrollment and before nucleos(t)ide analogue administration using a commercially available polyacrylamide-gel (PAG) disk electrophoresis kit designed for use in humans (AlkPhor System, Jokoh Co. Ltd, Tokyo, Japan) using serum stored at −30°C.

Serum levels of intact parathyroid hormone (PTH) were measured using an electrochemiluminescence immunoassay (Roche Diagnostics K. K.), and urinary cross-linked N-telopeptide of type I collagen (NTX) was measured using an ELISA (Osteomark, Inverness Medical Innovations Inc., Waltham, MA, USA) at the time of enrollment.

ΔBAP, a sensitive marker reflecting the changes in bone metabolism, was calculated as follows: serum BAP concentration at the time of enrollment minus serum BAP concentration before administration of nucleos(t)ide analogues. ΔBAP was then compared with the length of nucleos(t)ide analogue therapy.

### 2.4. Other Markers in Serum and Urine

Levels of phosphate and creatinine were examined at the time of enrollment and before nucleos(t)ide analogue administration using serum stored at −30°C. Levels of urinary phosphate were examined at the time of enrollment.

### 2.5. Statistical Analysis

Data are presented as median values (range). The Mann-Whitney *U* test and Wilcoxon rank-sum test were used to analyze continuous variables. Fisher's exact test was used for analysis of categorical data. All tests of significance were two tailed, and a *P* value of <0.05 was considered significant. All statistical analyses were performed using STATISTICA for Windows version 6 (StatSoft, Oklahoma, USA).

## 3. Results

### 3.1. Characteristics of 19 Well-Matched Pairs in the ADV and Non-ADV Groups

The baseline characteristics of the 144 patients enrolled in the study are summarized in Table 1. There were no significant differences with respect to age, sex, duration of nucleos(t)ide analogue therapy, platelet count, serum ALT, serum albumin, total bilirubin at the time of enrollment, and serum creatinine level at the time of nucleos(t)ide analogue administration between the ADV and non-ADV groups (Table 2).

### 3.2. Markers in Serum and Urine at the Time of Enrollment

**Figure 1:**
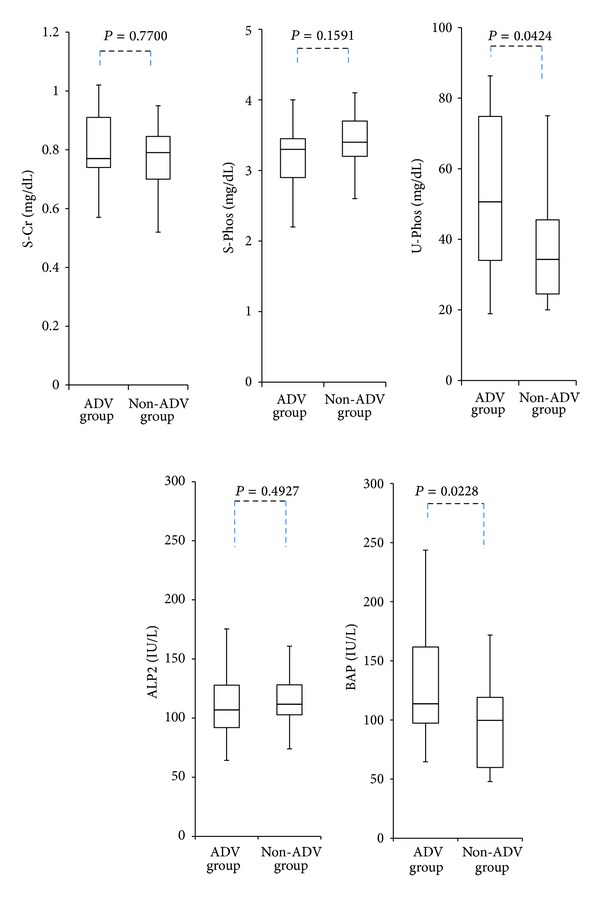
Serum creatinine, serum phosphate, urinary phosphate, serum ALP2, and BAP concentration at the time of at least 1 year or more after starting nucleos(t)ide analogue therapy in ADV group and non-ADV group (S-Cr; serum creatinine, S-Phos; serum phosphate, U-Phos; urinary phosphate, ALP; alkaline phosphatase, BAP; bone specific alkaline phosphatase).

There was no difference in serum creatinine concentration between the ADV and non-ADV groups at the time of enrollment. Serum phosphate concentration tended to be lower in the ADV group, although the difference was not statistically significant. In contrast, urinary phosphate concentration was significantly higher in the ADV group than in the non-ADV group (*P* = 0.0424). There was no significant difference between the ADV and non-ADV groups in the concentration of serum alkaline phosphatase (ALP) isoenzyme 2, which is liver specific; however, serum BAP concentration was significantly higher in the ADV group than in the non-ADV group (*P* = 0.0228) (Figure 1).

**Figure 2:**
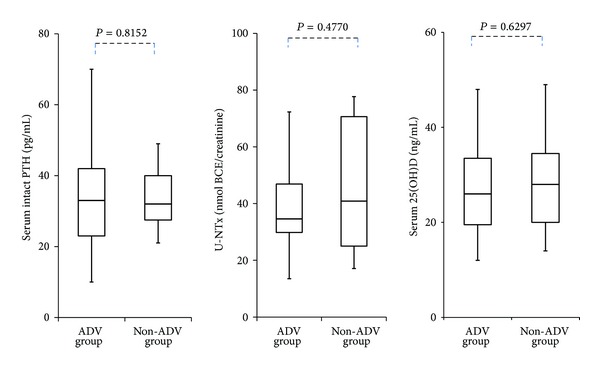
Serum intact PTH, U-NTx, and 25(OH)D concentrations at the time of at least 1 year or more after starting nucleos(t)ide analogue therapy in ADV group and non-ADV group (U-NTx: urinary N-telopeptide of type I collagen; intact PTH: intact parathyroid hormone; 25(OH)D:25-hydroxyvitamin D).

There were no significant differences in serum intact PTH, 25-hydroxyvitamin D, and urinary NTX concentrations between the ADV and non-ADV groups (Figure 2).

### 3.3. Changes in Serum Creatinine, Phosphate, and ALP Isoenzymes before and after Administration of Nucleos(t)ide Analogues

**Figure 3:**
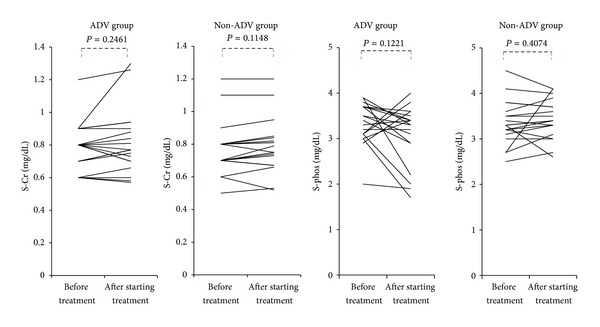
Transition of serum creatinine and phosphate concentrations from before treatment to at the time of at least 1 year or more after starting nucleos(t)ide analogue therapy in ADV group and non-ADV group (S-Cr; serum creatinine, S-Phos; serum phosphate, after starting treatment; at the time of at least one year or more after starting nucleos(t)ide analogue therapy).

**Figure 4:**
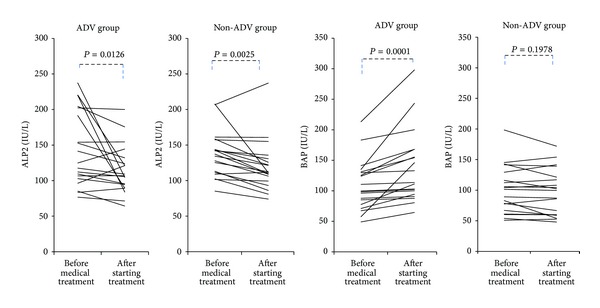
Transition of serum ALP2 and BAP concentrations from before medical treatment to at the time of at least 1 year or more after starting nucleos(t)ide analogue therapy in ADV group and non-ADV group.

There were no significant changes in serum creatinine or serum phosphate concentration with the administration of ADV or the other nucleoside analogues (LAM or ETV) (Figure 3). Serum ALP2 concentrations were significantly lower at the time of enrollment than before drug administration in both ADV group (*P* = 0.0126) and non-ADV group (*P* = 0.0025). Serum BAP was significantly higher at the time of enrollment than before nucleos(t)ide analogue administration in the ADV group (*P* = 0.0001). There was no significant change in serum BAP concentration in the non-ADV group (Figure 4).

### 3.4. Correlation between the Duration of Nucleos(t)ide Analogues Therapy and the Change in Serum ALP Isoenzyme Component

**Figure 5:**
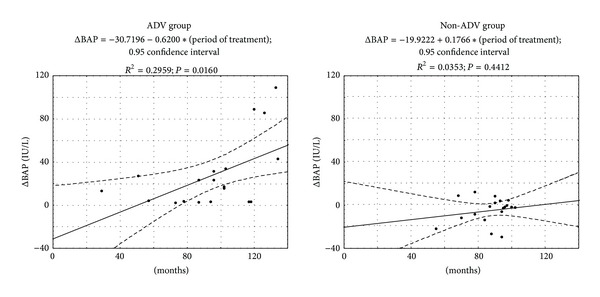
Relation between period of nucleos(t)ide analogue therapy and ΔBAP. ΔBAP; (serum BAP concentration at the time of at least 1 year or more after starting nucleos(t)ide analogue therapy)-(serum BAP concentration before treatment).

Although there was no correlation between the length of nucleoside analogue therapy and ΔBAP in the non-ADV group, there was a significant positive correlation between the period of ADV therapy and ΔBAP in the ADV group (*R* ^2^ = 0.2959; *P* = 0.0160, *R*: correlation coefficient) (Figure 5).

## 4. Discussion

In the present study, we found that serum BAP concentration was significantly elevated after the administration of ADV for more than 1 year. This finding was not observed after the administration of the other nucleoside analogues. Because serum BAP concentration reflects bone metabolism and is increased in osteomalacia, this finding suggests a tendency for subclinical osteomalacia in patients using ADV. However, we did not observe significant differences between the ADV and non-ADV treatment groups with respect to intact serum PTH concentration, 25-hydroxyvitamin D, and urinary NTX concentration, which are important markers of hyperparathyroidism, osteomalacia, and osteoporosis, respectively. This result may have been obtained because these bone turnover markers were examined in patients who did not develop symptomatic renal impairment or osteomalacia related to ADV treatment in this study.

A study evaluating long-term ADV therapy reported that 5% of the patients who received therapy up to 5 years had a slight elevation in serum creatinine concentration [24]. Although renal impairment is one of the most important side effects of ADV, we did not find a significant elevation in serum creatinine concentration among patients in that treatment group. However, we did observe that serum phosphate concentration tended to be lower and urinary phosphate concentration was significantly higher at the time of enrollment (≥1 year after starting ADV therapy) than before starting nucleos(t)ide analogue therapy. Therefore, it was difficult to detect potential renal dysfunction on the basis of elevation in serum creatinine concentration alone. According to a recent report, the maximal reabsorption of phosphate in the renal tubules and serum phosphate concentration were low in several patients receiving ADV; however, after changing therapy to ETV, serum phosphate concentration improved [25]. In the present study, urinary phosphate concentration was significantly higher in the ADV group than in the non-ADV group, although there was no significant difference in serum phosphate concentration. These findings may be attributed to the increase in urinary phosphate that occurs before a decrease in serum phosphate. Therefore, monitoring for an increase in urinary phosphate may be critical for earlier detection of osteomalacia caused by renal tubular impairment. 

Given that BAP is a bone turnover marker, we examined change in serum BAP concentration to determine the potential risks of osteomalacia caused by renal tubular impairment. Serum BAP concentration increased significantly after administration of ADV for >1 year. However, this finding was not observed after administration of the other nucleoside analogues. Therefore, osteomalacia caused by renal tubular impairment may be a unique side effect of ADV, especially after long-term use of ADV.

ADV plus LAM combination therapy is very useful in patients with LAM-resistant chronic HBV infection, and it is recommended as the first-line therapy for LAM-resistant disease in Japan [26]. However, because the criteria for discontinuation of nucleos(t)ide analogue therapy are not clear, long-term ADV plus LAM combination therapy is required in these patients to avoid risk of relapse. Long-term ADV plus LAM combination therapy carries a potential risk of renal impairment and development of Fanconi syndrome [17]. However, in the present study, patients did not present with symptoms of Fanconi syndrome; this finding may indicate that ADV plus LAM combination therapy is fairly safe for almost all patients if appropriate monitoring is provided for adverse effects of ADV.

Our results suggest that compared to serum creatinine and/or phosphate concentration, increases in concentrations of urinary phosphate and serum BAP are more useful indicators for early identification of renal tubular impairment. Further, serum BAP concentration increased before serum phosphate decreased; this finding may indicate that serum phosphate was being released from the bones, even though it was being lost through the kidneys. In addition, ΔBAP, a sensitive maker for detecting bone metabolism abnormalities, increased with the duration of ADV plus LAM therapy. Therefore, the risk of osteomalacia may increase with long-term use of ADV.

A previous report suggested that dose reduction of ADV to 10 mg every other day leads to improvement of renal function without compromising treatment efficacy [27]. In our experience, serum creatinine concentration increased from 0.9 mg/dL to 1.3 mg/dL after the addition of ADV (10 mg/day) to LAM monotherapy (100 mg/day) in 1 patient; subsequently, after 3 months of reducing the dose of ADV to 10 mg every other day, serum creatinine concentration improved to 1.0 mg/dL. However, because there is no obvious standard procedure for dose reduction, further studies are required to establish when and how to reduce ADV dose for patients whose serum ALP and/or urinary phosphate concentrations increase after long-term administration of ADV.

Furthermore, 2 adult patients with acquired immunodeficiency syndrome presented with severe bone pain associated with TDF [28], which is also used for the treatment of chronic HBV infection-related liver disease in Japan and is associated with an increase in serum ALP [29]. Although the relatively high rate of ETV resistance is a concern [30], therapy with 1.0 mg of ETV per day may be considered when ADV and TDF are contraindicated in patients with LAM resistance because of treatment-induced renal impairment and osteomalacia. 

Nucleos(t)ide analogue therapies are very useful for the treatment of chronic HBV infection-related liver disease, have few adverse effects with subjective symptoms, and are often used in long-term treatment. However, patients receiving these therapies should be supervised and monitored carefully for the early detection of adverse effects. Periodic measurement of serum BAP may be helpful in the early detection of osteomalacia in the absence of elevation of serum creatinine concentration.

## 5. Conclusions

In conclusion, examination of serum BAP concentration is useful for predicting osteomalacia caused by renal impairment in the absence of subjective symptoms and is essential for the establishment of adequate measures for determining the continuation of nucleos(t)ide analogue therapy for chronic HBV infection-related liver disease.

## Figures and Tables

**Table 1:** Baseline clinical characteristics of 144 patients received nucleos(t)de analogues with chronic HBV-infected liver disease at the time of enrollment.

	Median (range)		*P* value
	ADV group (*n* = 20)	Non-ADV group (*n* = 124)	
At the time of enrollment			
Age (yr)	52 (29–78)	55 (28–83)	0.8368
Sex (male/female)	15/5	82/42	0.5974
HBV genotype (A/B/C/F/n.d)	2/2/12/1/3	3/26/65/0/30	0.8671
HBV DNA (log_10_ copies/mL)	2.1 > (negative–3.0)	2.1 > (negative–6.2)	0.1896
Platelet count (×10^3^/m^3^)	16.5 (10.2–31.0)	16.8 (3.4–31.5)	0.1987
ALT (IU/L)	26 (10–60)	20 (8–124)	0.1117
Alb (g/dL)	4.4 (4–5.3)	4.4 (1.8–5.2)	0.9154
Cirrhosis (presence/absence)	5/15	30/94	0.8392
At the time of starting ADV or ETV			
Serum creatinine (mg/dL)	0.8 (0.6–1.2)	0.76 (0.52–1.30)	0.3853
Follow-up duration (month)	103 (37–141)	40 (12–152)	8.1*E* − 09
Duration of nucleos(t)ide analogue therapy (month)	103 (37–141)	34 (12–117)	9.8*E* − 10
Duration of adefovir administration (month)	73 (12–107)		
Propensity score	0.197 (0.068–0.553)	0.108 (3.49*E* − 05–0.514)	0.0018

ADV: adefovir dipivoxil; non-ADV: lamivudine or entecavir.

**Table 2:** Clinical characteristics of 19 well-matched pairs of ADV group and non-ADV group at the time of enrollment obtained by propensity score.

	Median (range)		*P* value
	ADV group (*n* = 19)	Non-ADV group (*n* = 19)	
At the time of enrollment			
Age (yr)	42 (29–68)	45 (34–68)	0.8940
Sex (male/female)	14/5	14/5	0.7126
HBV genotype (A/B/C/F/n.d)	1/3/11/1/3	1/4/10/0/4	0.7945
HBV DNA (log_10_ copies/mL)	2.1 > (negative–2.6)	2.1 > (negative–3.2)	0.2868
Platelet count (×10^3^/m^3^)	16.2 (10.2–31.0)	16.0 (9.8–28.3)	0.1675
ALT (IU/L)	26 (15–49)	25 (9–60)	0.8742
Alb (g/dL)	4.4 (4–5.3)	4.4 (3.9–5.3)	0.8714
Cirrhosis (presence/absence)	5/14	5/14	0.7126
Child Pugh score of cirrhosis cases	All 5 cases: 5	All 5 cases: 5	
At the time of starting ADV or ETV			
Serum creatinine (mg/dL)	0.8 (0.6–1.2)	0.78 (0.57–1.2)	0.7692
Follow-up duration (month)	96 (29–134)	90 (55–102)	0.1323
Duration of nucleos(t)ide analogue therapy (month)	96 (29–134)	90 (55–102)	0.1323
Duration of Adefovir administration (month)	73 (12–107)		
Propensity score	0.197 (0.068–0.553)	0.205 (0.067–0.514)	0.8267

ADV: adefovir dipivoxil; non-ADV: lamivudine or entecavir.
